# Thermal Conductivity Stability of Interfacial in Situ Al_4_C_3_ Engineered Diamond/Al Composites Subjected to Thermal Cycling

**DOI:** 10.3390/ma15196640

**Published:** 2022-09-24

**Authors:** Ning Li, Jinpeng Hao, Yongjian Zhang, Wei Wang, Jie Zhao, Haijun Wu, Xitao Wang, Hailong Zhang

**Affiliations:** 1 State Key Laboratory for Advanced Metals and Materials, University of Science and Technology Beijing, Beijing 100083, China; 2 Beijing Institute of Structure and Environment Engineering, Beijing 100076, China; 3 State Key Laboratory for Mechanical Behavior of Materials, Xi’an Jiaotong University, Xi’an 710049, China; 4 Collaborative Innovation Center of Steel Technology, University of Science and Technology Beijing, Beijing 100083, China; 5 Shandong Provincial Key Laboratory for High Strength Lightweight Metallic Materials, Advanced Materials Institute, Qilu University of Technology (Shandong Academy of Sciences), Jinan 250014, China

**Keywords:** diamond/Al composite, thermal cycling, interfacial structure, thermal stability

## Abstract

The stability of the thermal properties of diamond/Al composites during thermal cycling is crucial to their thermal management applications. In this study, we realize a well-bonded interface in diamond/Al composites by interfacial in situ Al_4_C_3_ engineering. As a result, the excellent stability of thermal conductivity in the diamond/Al composites is presented after 200 thermal cycles from 218 to 423 K. The thermal conductivity is decreased by only 2–5%, mainly in the first 50–100 thermal cycles. The reduction of thermal conductivity is ascribed to the residual plastic strain in the Al matrix after thermal cycling. Significantly, the 272 μm-diamond/Al composite maintains a thermal conductivity over 700 W m^−1^ K^−1^ after 200 thermal cycles, much higher than the reported values. The discrete in situ Al_4_C_3_ phase strengthens the diamond/Al interface and reduces the thermal stress during thermal cycling, which is responsible for the high thermal conductivity stability in the composites. The diamond/Al composites show a promising prospect for electronic packaging applications.

## 1. Introduction

With the ongoing breakthrough of process technology, electronic devices are integrating more components, and the power density is reaching a very high level [1,2]. High-power electronic devices are subjected to heat dissipation and are difficult to advance further due to the low thermal conductivity (TC) of traditional thermal management materials [3]. Diamond particles reinforced Al matrix (diamond/Al) composite enjoys high TC, tunable coefficient of thermal expansion (CTE), and suitable mechanical properties, and is acting as an ideal material for heat dissipation of high-power electronics [4,5,6,7]. The diamond/Al composites with high TC and light specific weight provide a desirable candidate for thermal management applications in the field of lightweight-vehicle and aerospace engineering.

Currently, the bonding strength of the diamond/Al interface is improved to acquire high TC in diamond/Al composites by optimizing preparation parameters or introducing a third phase at the interface. On one hand, Wang et al. [8] strengthened the interfacial bonding by controlling the cooling process, and the TC of the diamond/Al composite was enhanced from 321 to 606 W m^−1^ K^−1^. Zhang et al. [7] fabricated a diamond/Al composite with 763 W m^−1^ K^−1^ by adjusting the infiltration temperature and pressure of gas pressure infiltration. On the other hand, Guo et al. [9] enhanced the TC of the diamond/Al composite from 489 to 532 W m^−1^ K^−1^ by introducing SiC at the interface. Che et al. [10] prepared a diamond/Al composite with 650 W m^−1^ K^−1^ by employing TiC-coated diamond fillers. With the two routes, the TC of diamond/Al composites reached a high level of 500–770 W m^−1^ K^−1^ [4,5,6,7,8,9,10,11,12,13,14,15,16,17].

The interfacial Al_4_C_3_ phase in situ generated in diamond/Al composites contributes to the well-bonded interface and pushes the TC to a remarkably high level of 763 W m^−1^ K^−1^ [7], much higher than the diamond/Al composites modified via introducing other third phases at the interface. However, the role of Al_4_C_3_ in improving the TC of the diamond/Al composites is still a debate, especially for the thermal stability of the composites. Kondakci and Solak [18] reported that the diamond/Al composite shows low thermal durability and moisture resistance, owing to the unstable Al_4_C_3_ phase, and then presents the decline of TC. However, Monje et al. [19] argued that the diamond/Al composite containing the interfacial Al_4_C_3_ phase can maintain a stable and high TC after the composite is kept in a 100% relative humidity environment at 343 K for 500 h, and they indicated that the diamond/Al composite containing more interfacial Al_4_C_3_ phase shows a stable and high TC after the composite is subjected to 1000 thermal cycles from 298 to 423 K. Wang et al. [20] suggested that the interfacial Al_4_C_3_ phase tightly bonds the diamond particles and the Al matrix even after 200 thermal cycles. Indeed, the influence of the interfacial Al_4_C_3_ phase on diamond/Al composites subjected to thermal cycling is not clear, and thus more researches are needed to achieve a comprehensive understanding of the arguable Al_4_C_3_. The thermal stability of electronic packaging materials is important for applications with varying temperatures.

In this study, the in situ Al_4_C_3_ engineered diamond/Al composites with different diamond particle sizes are subjected to thermal cycling from 218 to 423 K. The interfacial structure and TC of the diamond/Al composites are explored with respect to thermal cycling. The evolution of the TC is explicated by the discrete in situ Al_4_C_3_ particles as well as the residual plastic strain in the composites calculated by a theoretical model. The study highlights the interfacial discrete in situ Al_4_C_3_ in maintaining the excellent stability of TC in the diamond/Al composites and explains the correlation between TC evolution and plastic strain in the diamond/Al composite subjected to thermal cycling.

## 2. Experiment Section

### 2.1. Sample Fabrication

Synthetic diamond single-crystals with diameters of 66 μm and 272 μm (Henan Huanghe Whirlwind Co., Changge, China) and Al bulks (99.99 wt%, Baotou Aluminum Group Co., Baotou, China) were employed as the reinforcement and matrix, respectively. The TC of the diamond particles was derived according to the equation *λ* = 2200 − 3.27[N] [21], where *λ* is the TC and [N] is the nitrogen concentration in the diamond particles. The detailed properties of the diamond particles are displayed in Table 1.

**Figure 1:**
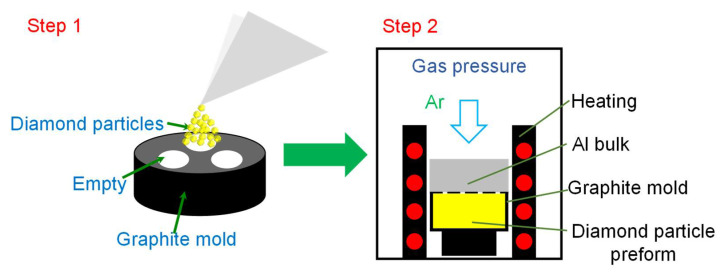
Schematic illustration of the preparation of the diamond/Al composites by gas pressure infiltration.

The diamond/Al composites were fabricated by a homemade gas pressure infiltration facility. The schematic illustration of the preparation of the diamond/Al composites is displayed in Figure 1. The diamond particles were densely vibrated into a graphite mold to form diamond particle preform, and then the graphite mold was covered by an Al bulk. The Al bulk was heated to 1073 K and maintained for 30 min before an Ar gas pressure of 1.0 MPa was applied for infiltration. The infiltration was performed at 1073 K for 20 min under the pressure of 1.0 MPa. Then, the sample was cooled down to room temperature in the chamber. The diamond volume fraction of the 66 μm and 272 μm-diamond/Al composites were 58.2% and 59.2%, respectively.

### 2.2. Characterization of the Diamond/Al Composites

The phase component of the prepared diamond/Al composites was identified by X-ray diffraction (XRD, Rigaku DMAX-RB, Tokyo, Japan) with Cu K_α_ radiation. A field-emission scanning electron microscope (SEM, ZEISS SUPRA 55, Oberkochen, Germany) was applied to analyze the surface morphology of the diamond particles as well as the fracture surface of the diamond/Al composites. For exposing the diamond particle surfaces, the Al matrices were partly removed from the diamond/Al composites by electro-chemical etching using a 10 vol.% HNO_3_ solution. The interface structure of the diamond/Al composites was characterized by a scanning transmission electron microscope (STEM, JEOL ARM200F, Tokyo, Japan). The TEM lamellas of the composite were prepared by a dual beam focused ion beam (FIB, FEI Versa 3D Dualbeam, Hillsboro, WA, USA). 

**Figure 2:**
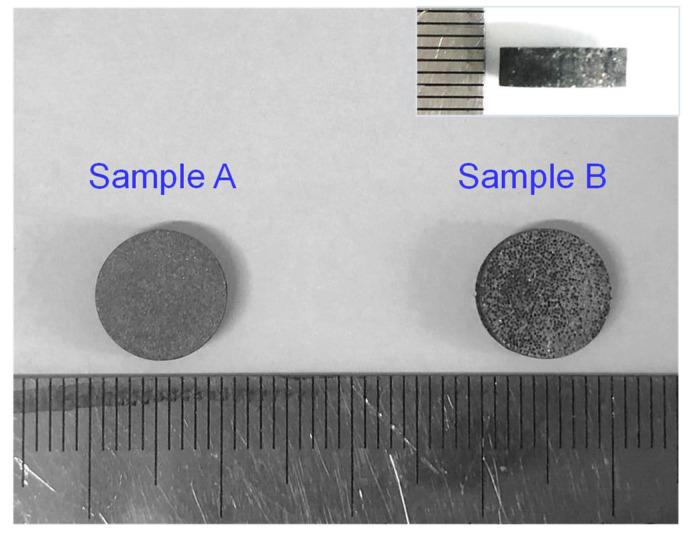
Optical images exhibiting the dimensions of the diamond/Al composite samples.

The TC of the diamond/Al composites was calculated by the equation *λ* = *αρC*_p_, where the *α* is thermal diffusivity, *ρ* is sample density, and *C*_p_ is specific heat capacity. The thermal diffusivity was measured with a laser flash equipment with a testing error less than 3% (LFA467, Netzsch, Selb, Germany) using a standard sample with the dimensions of Φ10 mm × 3 mm, as shown in Figure 2. Sample A and sample B refer to the 66 μm- and 272 μm-diamond/Al composites, respectively. The sample density *ρ* was examined by the Archimedes method. The specific heat capacity *C*_p_ was derived from the rule of mixture based on the mass fraction of each component. 

### 2.3. Thermal Cycling Test

The thermal cycling test was performed in a thermal shock test chamber (TSD-101-W, ESPEC, Osaka, Japan). To evaluate the thermal cycling properties of the diamond/Al composites accurately, the samples were packaged into a quartz tube and filled with Ar gas to prevent oxidation during the thermal cycling test. To simulate the working circumstance in electronic packaging applications, the thermal cycling test was subjected to the JESD22-A104C standard [22], and the H test condition with a temperature range of 218 to 423 K was selected. According to the standard, the dwell time could be selected as 1, 5, 10, or 15 min. The samples were held in the high temperature and the low temperature chambers for 10 min each. According to the standard, the cycling rate of 1–3 cycles per hour was regulated. The switching time from the high temperature chamber to the low temperature chamber was less than 10 s, and the cycling rate was around 3 cycles per hour, which meets the requirements of the standard. The thermal cycle number was not specified in the standard. The thermal cycle number of 200 in reference [20] was referenced. After 50, 100, 150, and 200 thermal cycles, the TC of the samples was measured at 298 K.

## 3. Result and Discussion

### 3.1. Interfacial Structure of the Diamond/Al Composites Subjected to Thermal Cycling

**Figure 3:**
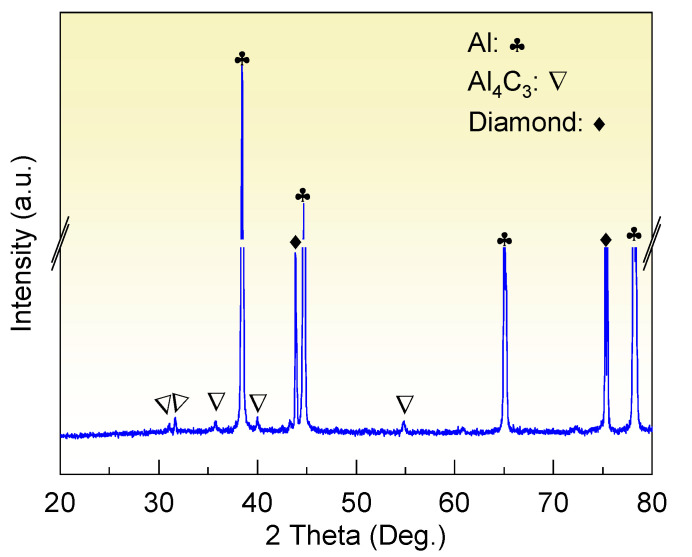
XRD pattern of the diamond/Al composite.

**Figure 4:**
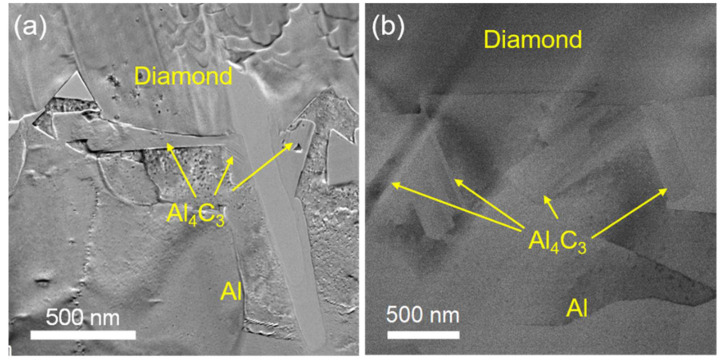
TEM images of the discrete in situ Al_4_C_3_ at the interface in the diamond/Al composites: (**a**) diamond (100) surface and (**b**) diamond (111) surface.

**Figure 5:**
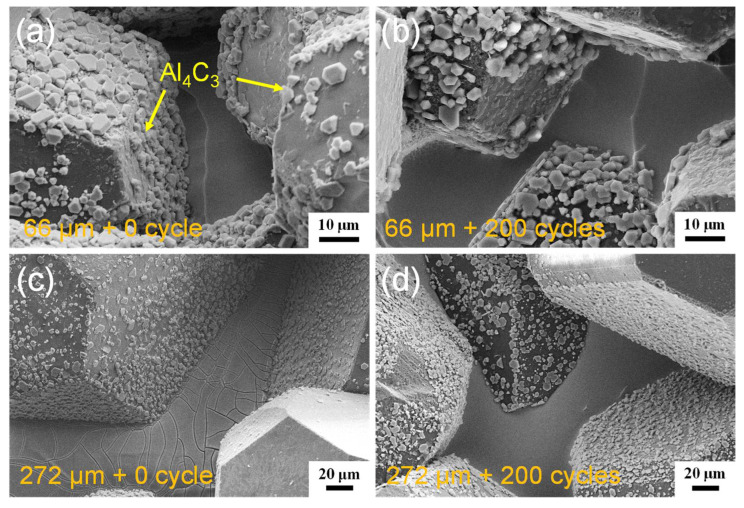
Interface morphologies of the diamond/Al composites with different diamond particle sizes before thermal cycling: (**a**) 66 μm and (**c**) 272 μm, and after 200 thermal cycles; (**b**) 66 μm and (**d**) 272 μm.

The XRD analysis in Figure 3 suggests that the diamond/Al composite is composed of diamond, Al, and Al_4_C_3_. The characteristic peaks at 31.1°, 31.7°, 35.7°, 40.1°, and 55.0° indicate the generation of the Al_4_C_3_ phase in the diamond/Al composites. The interfacial structure of the diamond/Al composites was characterized via STEM before thermal cycling. As displayed in Figure 4a,b, the in situ formed Al_4_C_3_ phase is discrete on both diamond particle (100) and (111) surfaces, which helps to connect the diamond particle and the Al matrix closely owing to a pinning effect. The discrete in situ Al_4_C_3_ phase grows from the diamond particle surfaces and then pierces into the Al matrix, which creates an interlock structure and thus strengthens the interface. The surface morphologies of the diamond particles in the diamond/Al composites before and after 200 thermal cycles are compared in Figure 5. The discrete morphology of the Al_4_C_3_ phase on the diamond particles does not change after thermal cycling. The low thermal cycling temperature cannot provide enough energy for the growth of Al_4_C_3_. The unchanged morphology of the discrete Al_4_C_3_ phase on the diamond particle surfaces indicates that the strong interface is maintained after thermal cycling. It is noted that more Al_4_C_3_ phase is formed on the larger diamond particle surfaces when comparing Figure 5a,b with Figure 5c,d. This suggests that stronger interface is formed in the diamond/Al composite with larger diamond particles.

### 3.2. Fracture Surfaces of the Diamond/Al Composites Subjected to Thermal Cycling

**Figure 6:**
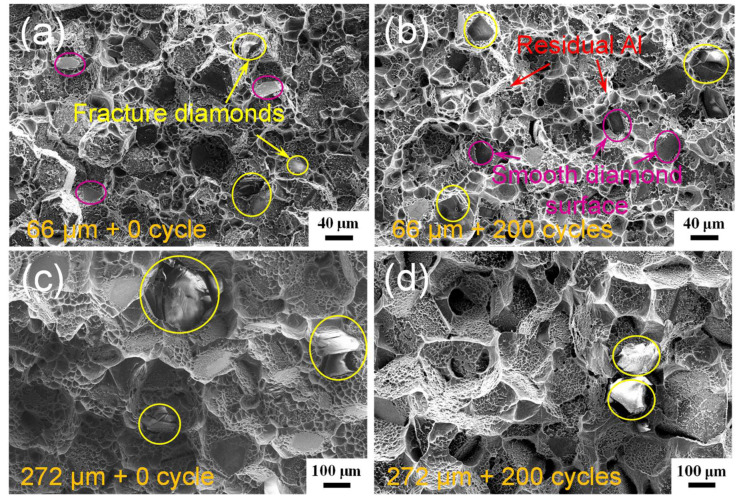
Fracture surfaces of the diamond/Al composites with different diamond particle sizes before thermal cycling: (**a**) 66 μm and (**c**) 272 μm, and after 200 thermal cycles: (**b**) 66 μm and (**d**) 272 μm.

Figure 6 displays the fracture surfaces of the diamond/Al composites before and after 200 thermal cycles. The fractured diamond particles, the residual Al matrix on the diamond particle surfaces, and the Al dimples are clearly observed on the fracture surfaces of the diamond/Al composites. This reveals that the fracture starts from the Al matrix or the diamond fillers, and that the bonding strength of the diamond/Al interface is higher than the fracture strength of pure Al and synthetic diamond containing defects. The fracture surface morphology of the diamond/Al composites does not change after thermal cycling. Wang et al. [20] reported that the interfacial gap emerges at the diamond (111)/Al interface due to the “net” (no Al_4_C_3_) interface after 200 thermal cycles. In this study, both the diamond particle (100) and (111) surfaces are densely covered with Al_4_C_3_ phase, as shown in Figure 5a,c. After 200 thermal cycles, both the diamond (100)/Al and diamond (111)/Al interfaces are tightly bonded and interfacial gaps are not observed, as shown in Figure 6b and d. The diamond/Al composites can withstand the thermal cycling, which is a result of the interfacial Al_4_C_3_ phase. First, the Al_4_C_3_ phase in situ grows on diamond particle surfaces and realizes a chemical bonding, forming the strong diamond/Al interface. Second, Al_4_C_3_ has a CTE of 8.0 × 10^−6^ K^−1^ [23] in between diamond and Al, which reduces the generation of thermal stress at the interface during heating and cooling. Third, the discrete morphology of the Al_4_C_3_ phase further releases the concentration of interfacial thermal stress. Comparing the diamond/Al composites with small diamond particles (Figure 6a,b), more fractured diamond particles and less smooth diamond particle surfaces are observed in the diamond/Al composite with large diamond particles (Figure 6c,d), indicating a stronger interface. This is consistent with the more Al_4_C_3_ generated on the large diamond particle surfaces, as presented in Figure 5c,d.

### 3.3. Thermal Conductivity of the Diamond/Al Composites Subjected to Thermal Cycling

**Figure 7:**
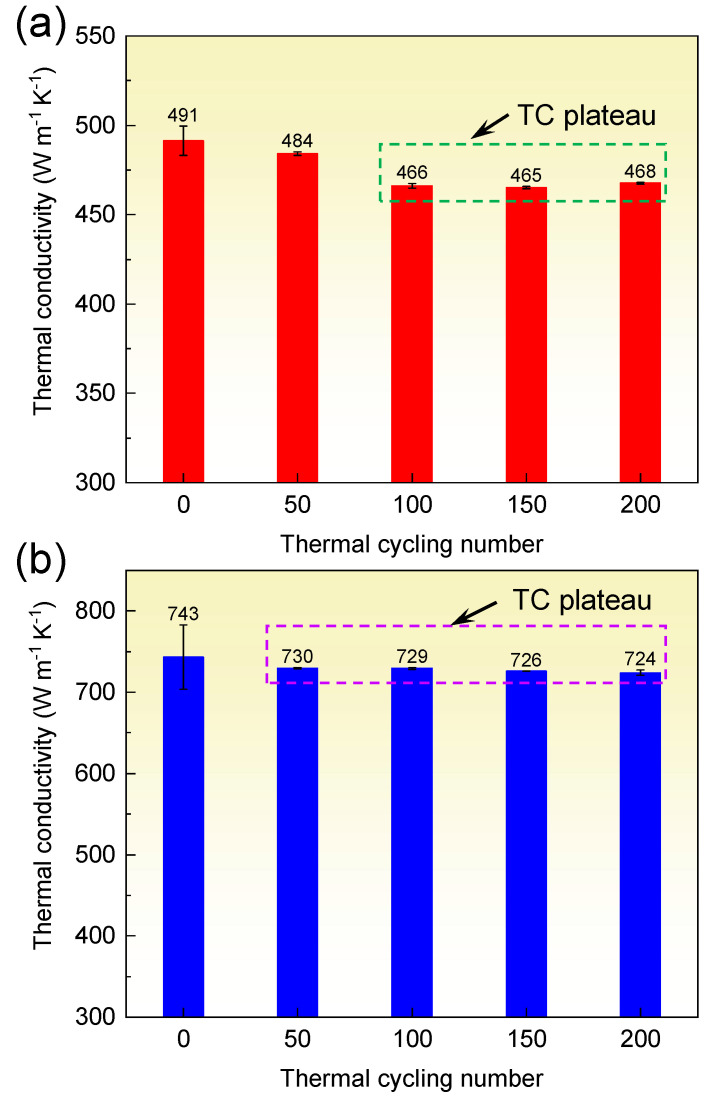
Thermal conductivity of the diamond/Al composites with different diamond particle sizes versus thermal cycle: (**a**) 66 μm and (**b**) 272 μm.

As electronic packaging materials, the diamond/Al composites must endure high and low temperature cycling and preserve acceptable thermal properties. The stability of TC in the diamond/Al composites subjected to thermal cycling is important for their application. The TC of the diamond/Al composites after 0, 50, 100, 150, and 200 thermal cycles is displayed in Figure 7. The TC of the diamond/Al composites declines similarly when the thermal cycling number rises. The TC still remains high value after thermal cycling. Remarkably, the TC of the 272 μm-diamond/Al composite keeps a rather high value of 724 W m^−1^ K^−1^ after 200 thermal cycles. The declined percentages of TC of the 66 μm- and 272 μm-diamond/Al composites are only 4.7% and 2.6% after 200 thermal cycles, respectively. The reductions are much smaller than the referenced results. Wang et al. [20] found that after 200 thermal cycles, the TC of the diamond/Al composite is reduced from 606 to 547 W m^−1^ K^−1^ with a reduction of 9.7%. Bai et al. [24] suggested that the TC of the unmodified diamond/Cu composite is dramatically dropped from 112 to 92 W m^−1^ K^−1^ with a reduction of 17.9% after just 25 thermal cycles, because the weak diamond/Cu interface by van der Waals force is easily destroyed by the thermal stress. In the diamond/Al composites, however, the in situ generated Al_4_C_3_ phase creates a strong diamond/Al interface, which is able to endure the thermal stress. Thus, high TC is reserved in the diamond/Al composites even after 200 thermal cycles.

The TC of the diamond/Al composites were determined by the TC of the diamond particle and the Al matrix as well as the interfacial thermal conductance of the diamond/Al interface. Zhang et al. [25] suggested that the TC of diamond declines slightly from 1647 to 1631 W m^−1^ K^−1^ after heat treatment at 1273 K. It is thus believed that the TC of the diamond particles is not affected after thermal cycling from 218 to 423 K. As illustrated in Figure 5 and Figure 6, the interfacial structure in the diamond/Al composites does not change, and the interface is still well-bonded after thermal cycling. It was rationally deduced that the interfacial thermal conductance of the diamond/Al interface does not change after thermal cycling. Accordingly, the decrease of TC in the composites results from the decrease of TC in the Al matrix. According to the Wiedemann–Franz law, the TC of metal scales with the electrical conductivity. The electrical resistivity, reciprocal of electrical conductivity, of metal is mainly determined by the scattering of defects like impurities, dislocations, and grain boundaries. The electrical resistivity is expressed as follows [26]:

(1) $$\rho_{\mathrm{total}}{=\rho }_{\mathrm{impurities}}+\rho_{\mathrm{dislocations}}+\rho_{\mathrm{grain}\;\mathrm{boundaries}}+\;\cdots$$

where the $\rho_{\mathrm{total}}$ is the total electrical resistivity. The $\rho_{\mathrm{impurities}}$, $\rho_{\mathrm{dislocations}}$, and $\rho_{\mathrm{grain}\;\mathrm{boundaries}}$ are the electrical resistivities caused by the scattering mechanisms of impurities, dislocations, and grain boundaries, respectively. The variation of electrical resistivity caused by the dislocations can be calculated as:

(2) $${\delta \rho }_{\mathrm{dislocations}}=\;\rho_{D}{\times \;N}_{D}$$

where $\rho_{D}$ is the varied value of resistivity per unit length of dislocation, and $N_{D}$ is the dislocation density. In the metal matrix composite, the dislocation density in the metal matrix improves significantly after thermal cycling [27,28]. As a result, the electrical resistivity of the Al matrix is enhanced according to Equations (1) and (2), and the TC of the Al matrix is lowered. Then, the TC of the diamond/Al composite is decreased. Moreover, the scattering of heat carriers at the diamond/Al interface is enhanced owing to the accumulation of the dislocations created by the concentration of interfacial thermal stress, which further hinders the heat transport at the diamond/Al interface and lowers the TC in the composites. To evaluate the decrease of TC in the Al matrix, a theoretical model [29] was employed to back-derive the TC of the Al matrix after thermal cycling. The results are shown in Table 2. Assuming a TC of 237 W m^−1^ K^−1^ for the Al matrix before thermal cycling, the TC of the Al matrix decreases apparently after thermal cycling. The decrease in TC of the Al matrix is reasonable in practice. Ye et al. [30] found that the TC of Cu declines linearly with the increasing tensile strain of Cu. The decrease in electrical conductivity of metals due to plastic strain has also been reported. Narutani and Takamura [31] suggested that the electrical conductivity of Ni decreases constantly as the dislocation density increases with plastic strain. Cho et al. [32] suggested that the electrical conductivity of the Al alloy drops as the plastic strain increases. The lowered electrical conductivity by plastic strain reduces the TC in metals according to the Wiedemann–Franz law. With the rising of the thermal cycling number, the plastic strain in the Al matrix increases, and the electrical conductivity of the Al matrix declines accordingly, which reduces the TC of the Al matrix.

The TC decline mainly happens after 50–100 thermal cycles, and then a smooth plateau is observed, as marked in Figure 7. The TC of the 66 μm-diamond/Al composite was reduced by 5.1% after 100 thermal cycles, and the TC of the 272 μm-diamond/Al composite was reduced by 1.7% after 50 thermal cycles. The phenomenon is consistent with referenced results [33] that the TC of SiC_p_/Al composites exhibits some smooth plateaus during thermal cycling. This could be explained by the increase of the dislocation density in the Al matrix. During the early stage of thermal cycling, the dislocation density increased rapidly in the neighborhood of the interface owing to the concentration of thermal stress. As the thermal cycling number rises, although the dislocation density increases constantly, the dislocation at the interface is almost saturated. As the thermal cycling number rises further, the dislocation density in the Al matrix increases again. It is worthy to note that the plastic deformation in the Al matrix is moderately reduced because the Al matrix is strengthened via the strain-hardening effect. Accordingly, the increase of dislocation density is gradually reduced. Since the TC of the Al matrix is tightly related to dislocation density, the variation of dislocation density explains the varied TC in the diamond/Al composites in Figure 7. The dislocation density depends on the residual plastic strain in the Al matrix, which will be discussed below.

### 3.4. Residual Plastic Strain in the Diamond/Al Composites Subjected to Thermal Cycling

The plastic strain (*ε_n_*) is defined as the dimension change of the sample before and after thermal cycling [34], which is important for the application of composites at variable temperatures. Here, the plastic strain is employed to address the stability of TC in the diamond/Al composites. The plastic strain is accumulated owing to plastic deformation of the Al matrix during thermal cycling, and the amplitude of plastic deformation rises as the thermal cycling number increases. In the literatures [19,20], some gaps are observed at the interface in diamond/Al composites after thermal cycling due to the weak interface bonding. In this study, since the gaps are not observed at the diamond/Al interface, the theoretical plastic strain that the interface should bear is calculated.

**Figure 8:**
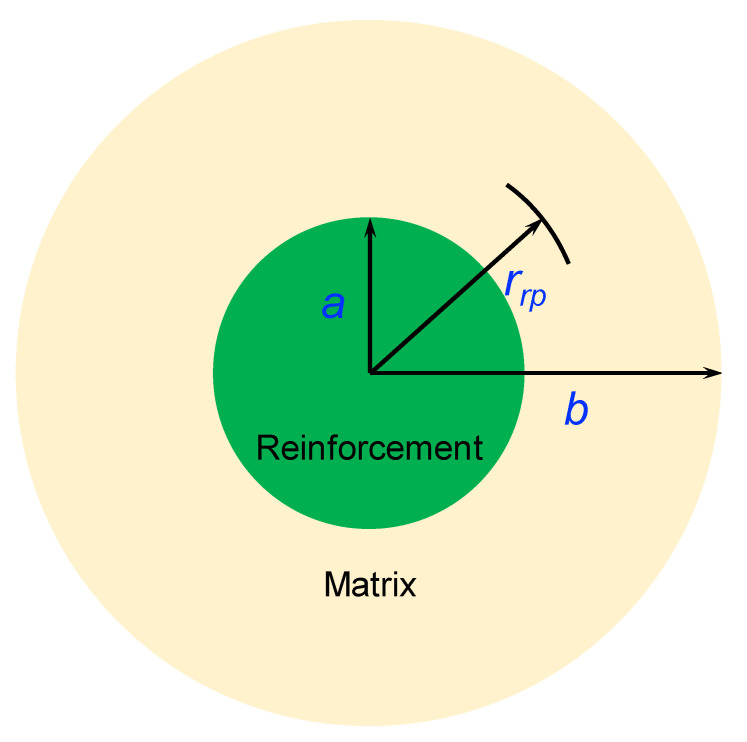
Schematic illustration of the representative volume element in the reinforcement/matrix composite.

Olsson et al. [35] suggested a theoretical model to analyze the plastic strain in metal matrix composites during thermal cycling. The composite is supposed to be composed of two concentric spheres with different materials. The hollow sphere represents the metal matrix and the inner sphere represents the reinforcement, as shown in Figure 8. The *a* and *b* refer to the radii of the reinforcement and the matrix, respectively. The representative volume element is a spherical unit with a radius *r*. Then, *a* < *r* < *b* refers to the matrix region, and 0 < *r* < *a* refers to the reinforcement region. The reinforcement volume fraction is thus calculated as ${\left(\frac{a}{b}\right)}^{3}$. For simplification, the following assumptions are necessary: (a) only the matrix performs the plastic deformation; (b) the matrix is an ideal elastic–plastic material; and (c) the matrix enjoys equal tensile and compressive yield strengths with no dependence on temperature. The plastic strain $\varepsilon_{n+1}^{\mathrm{eff}}$ at *r* (*a* < *r* < *b*) in the composite after *n* (*n* > 1) thermal cycles can be calculated by:

(3) $$\varepsilon_{n+1}^{\mathrm{eff}}{(r)=\varepsilon }_{1}(r)+\overset{n}{\underset{1}{\sum }}\varepsilon_{n}\left(r\right)$$

Taking no strain-hardening effect into consideration in the Al matrix, after *n* thermal cycles, the plastic strain $\varepsilon_{n}\left(r\right)$ is calculated to be:

(4) $$\varepsilon_{n}(r)=\frac{{2\sigma }_{y}{(1-v}_{m})\;}{E_{m}}\left\{\left[{\left(\frac{b}{r}\right)}^{3}\left(1+\frac{{cE}_{m}\left|\alpha_{r}{-\alpha }_{m}\right|(\left|\Delta T_{a}\right|-\Delta T)}{\sigma_{y}{(1-v}_{m})}\right)-1\right]+2(n-1)\;\left[{\left(\frac{r_{\mathrm{rp}}}{r}\right)}^{3}-\;1\right]\right\}\;\;\;\;\;a\;\leq \;r\;\leq {\;r}_{\mathrm{rp}}$$

where the *σ*_y_, *v*, *α*, and *E* represent the yield strength of the matrix, Poisson ratio, CTE, and Young’s modulus, respectively. The subscripts “m” and “r” refer to the matrix and reinforcement, respectively. The *c* represents the reinforcement volume fraction. The Δ*T*_a_ represents the temperature variation during thermal cycling. The Δ*T* and $r_{\mathrm{rp}}$ are the temperature variation of the full plasticity of the matrix and the reversed plastic region [35], respectively. When the thermal cycling number *n* > 1, the strain-hardening effect on the matrix is taken into consideration. The increasement of plastic strain for every thermal cycle [Δ$\varepsilon_{n}$(*r*) = $\varepsilon_{n+1}$(*r*) − $\varepsilon_{n}$(*r*)] is obtained by:

(5) $$\Delta \varepsilon_{n}(r)=\frac{{4\sigma }_{y}{(1-v}_{m})\;}{{(1+\beta )E}_{m}}\left[{\left(\frac{r_{\mathrm{rp}}}{r}\right)}^{3}-\;1\right]{\left(\frac{1-\beta \;}{1+\beta }\right)}^{n-1}\;\mathrm{with}\;\beta =\frac{{2H(1-v}_{m})\;}{E_{m}}$$

where *H* denotes the strain-hardening of the matrix. As deduced from Equations (3) and (5), the plastic strain $\varepsilon_{n}\left(r\right)$ approaches a limiting value, and the increasement of plastic strain $\Delta \varepsilon_{n}\left(r\right)$ approaches 0 when *n* → +∞. Subsequently, the plastic strain is inversely proportional to $r^{3}$. The effective strain distribution achieves the peak value at the interface. In other words, the interface in the diamond/Al composite bears the maximum plastic strain.

**Figure 9:**
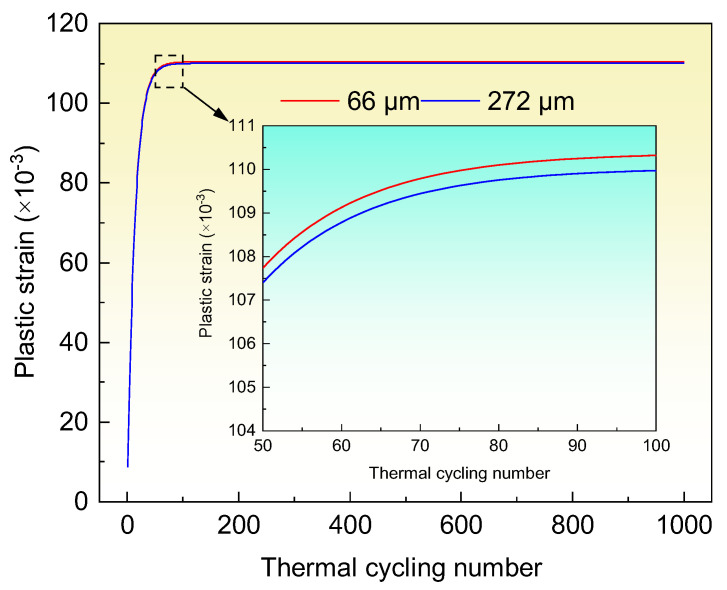
Calculated plastic strain in the diamond/Al composites with different diamond particle sizes after 1000 thermal cycles. The inset shows the calculated plastic strain during 50–100 thermal cycles.

The material parameters for calculating the plastic strain at the diamond/Al interface are shown in Table 3. Assuming the interfacial bonding of the diamond/Al composites is infinitely strong, the plastic strain at the interface can be calculated. Figure 9 shows the calculated plastic strain after 1000 thermal cycles. It is notable that the plastic strain improves as the thermal cycling number increases. The plastic strain rises dramatically in the first 50 thermal cycles, and then the increase drops during the 50–100 thermal cycles. When the thermal cycling number exceeds 100, the plastic strain approaches constant. The calculations of the plastic strain are in broad agreement with the TC variation in Figure 7. According to the Taylor equation [36], the flow stress *σ* and dislocation density $\rho_{d}$ present a positive relationship of *σ* ∝ $\sqrt{\rho_{d}}$. The dislocation density in metals increases with the increasing of the plastic strain in metals, as evidenced by Narutani and Takamura [31] and Cho et al. [32]. In the diamond/Al composites, the dislocation density in the Al matrix increases rapidly during the first 50 thermal cycles due to the large increase of plastic strain, which decreases the TC of the Al matrix. Thus, the TC of the 66 μm- and 272 μm-diamond/Al composites is apparently decreased. During the 50–100 thermal cycles, the small increase of the plastic strain implies that the dislocation density increases slowly, and the dislocation gradually saturates in the Al matrix. In the 66 μm-diamond/Al composite, the Al matrix is divided into numbers of small-scale units by the small diamond particles, and each Al unit has a high dislocation density due to the high plastic strain. This results in the decrease in TC of the Al matrix and therefore lowers the TC of the composite during the 50–100 thermal cycles, as shown in Figure 7a. While the Al unit in the 272 μm-diamond/Al composite is larger than that in the 66 μm-diamond/Al composite, the Al unit has a lower dislocation density because of the lower plastic strain. Thereby, the TC of the Al matrix is moderately affected, and the TC of the composite presents a stable value during the 50–100 thermal cycles, as shown in Figure 7b. After 100 thermal cycles, the plastic strain increases hardly, which implies that the dislocation density in the Al matrix is at a stable level. Accordingly, the TC of the Al matrix is unchanged, and the TCs of both the diamond/Al composites remain stable.

It can be noted that the diamond/Al composites with different diamond particle sizes present various TC declines. On one hand, the plastic strain in the diamond/Al composites varies with the diamond particle size. After 200 thermal cycles, the plastic strains in the 66 μm- and 272 μm-diamond/Al composites are 0.11038 and 0.11004, respectively. The 272 μm-diamond/Al composite exhibits a plastic strain slightly lower than the 66 μm-diamond/Al composite. This could be ascribed to the small difference of diamond content in the two composites. Assuming the diamond/Al composite is dense, and the volume variation is mainly caused by the Al matrix with large CTE, the volume change (Δ*V*_c_) in the composite can be estimated by [40]:

(6) $${\Delta V}_{c}=\;{3V}_{m}{(\alpha }_{m\;}{-\;\alpha }_{r})\Delta T$$

where the *α*_m_ and *α*_r_ are the CTE of the Al and diamond, respectively. The *V*_m_ denotes the volume fraction of the Al matrix. The Δ*T* refers to the temperature change. Taking the reference data of *α*_m_ = 23.4 × 10^−6^ K^−1^ and *α*_r_ = 1.0 × 10^−6^ K^−1^ from Table 3, and the measured data of *V*_m_ = 41.8% for the 66 μm-diamond/Al composite and *V*_m_ = 40.8% for the 272 μm-diamond/Al composite, the volume changes of the 66 μm- and 272 μm-diamond/Al composites are calculated to be 0.98% and 0.96%, respectively. The high diamond content reduces the volume variation of the composite during thermal cycling, and a low plastic strain occurs. Thus, the TC of the Al matrix in the 272 μm-diamond/Al composite is less reduced. On the other hand, the 66 μm-diamond/Al composite exhibits weaker interface than the 272 μm-diamond/Al composite, as illustrated in Section 3.1 and 3.2. This could further reduce the interfacial thermal conductance and then lower the TC of the 66 μm-diamond/Al composite. Accordingly, the 66 μm-diamond/Al composite shows a larger TC decline.

In diamond/Al composites, the Al matrix is imposed a tensile stress after fabrication owing to the large gap between the CTE of diamond and the CTE of Al. During the heating process of thermal cycling, the residual tensile stress is helpful to the expansion of the Al matrix. As the temperature increases, the Al matrix expands continuously and the residual tensile stress is released gradually. As the temperature further increases, the stress state in the Al matrix switches from tensile stress to compressive stress owing to the mismatched CTE between the diamond and Al. When the compressive stress surpasses the yield strength of the Al matrix, the plastic deformation occurs, and the compressive stress is relaxed to some extent. Although the reverse stress on the diamond particles is the same level as that on the Al matrix, the deformation of the diamond particles is negligible because of the huge modulus and strength. During the following cooling process of thermal cycling, the compressive stress in the Al matrix is gently released until the tensile stress is created again. However, the tensile stress is unable to cause a secondary plastic deformation in the Al matrix, since the strength of the Al matrix has been improved via the strain-hardening effect, and the Al matrix has higher strength at lower temperature. Thus, the plastic deformation is reserved after thermal cycling, namely thermal residual strain [41,42]. During thermal cycling, the strength of the Al matrix is strengthened constantly by the strain-hardening effect, which decreases the plastic deformation in the Al matrix and then reduces the increment of the residual strain in every thermal cycle. Consequently, the plastic strain in diamond/Al composites reaches a maximum value at a certain thermal cycling number.

Moreover, the well-bonded diamond/Al interface avoids the defects of gaps at the interface that could otherwise be produced during thermal cycling. In the literature, the plastic strain undermines the interface owing to the weak interfacial bonding, and then the TC of the composites drops greatly after thermal cycling [24]. In this study, the plastic strain could not wreck the interface after 200 thermal cycles, and the TC of the diamond/Al composites drops slightly. The interface can sustain a plastic strain up to 0.11038, indicating the strong interface again.

## 4. Conclusions

The diamond/Al composites with different diamond particle sizes were prepared via gas pressure infiltration and were subjected to 200 thermal cycles. The stability of thermal conductivity in the diamond/Al composites was addressed. The interfacial structure was characterized, and the plastic strain was calculated to explain the evolution of thermal conductivity in the diamond/Al composites. The main conclusions are presented as follows.

(1) The thermal conductivity of the diamond/Al composites with different diamond particle sizes declines as the thermal cycling number increases, and the thermal conductivity declines mildly in a range of 2–5% after 200 thermal cycles. The 272 μm-diamond/Al composite still maintains a high thermal conductivity over 720 W m^−1^ K^−1^ after thermal cycling.
(2) The strong interface created by the discrete in situ Al_4_C_3_ phase withstands the thermal stress during thermal cycling, which is responsible for the excellent stability of thermal conductivity in the diamond/Al composites. The decrease of thermal conductivity of the Al matrix is the main reason for the decline of the thermal conductivity of the composites.
(3) The decrease in the thermal conductivity of the Al matrix is explained by the dislocation density and is well supported by the calculations of plastic strain in the Al matrix during thermal cycling.
(4) The diamond/Al composites show promising application as an electronic packaging material because of their high thermal conductivity and excellent stability of thermal conductivity.

## Figures and Tables

**Table 1:** Material properties of the diamond particles.

Diamond Particle Size (μm)	Nitrogen Concentration [N] (ppm)	Thermal Conductivity *λ* (W m^−1^ K^−1^)
66	189	1582
272	129	1778

[N] was measured by a nitrogen oxygen meter.

**Table 2:** Calculated thermal conductivity of the Al matrix after thermal cycling.

Sample	Diamond Particle Size (μm)	Thermal Conductivity of the Al Matrix (W m^−1^ K^−1^)	Reduction (%)
A	66	208	12.2
B	272	222	6.3

**Table 3:** Material properties of diamond and Al used in the calculation of plastic strain.

Material	*v*	*E* (GPa)	*α* ( × 10^−6^ K^−1^)	H (GPa)	*σ*_y_ (MPa)
Al	0.33 [34]	72	23.4 [37]	2 [34]	30 [34]
Diamond	0.07 [38]	1141 [38]	1.0 [39]	-	elastic

## Data Availability

The data presented in this study are available on request from the corresponding author.
